# Bright lights, big synapses: fluorescent proteins let neurons shine

**DOI:** 10.1186/s12915-023-01808-7

**Published:** 2024-02-05

**Authors:** Brian D. Ackley

**Affiliations:** 5004 Haworth Hall, 1200 Sunnyside Ave, Lawrence, KS 66045 USA

## Abstract

**The availability of fluorescent proteins to visualize neurons and synapses has revolutionized our understanding of these key structures in brains. Here, I will discuss the new insights gleaned from the use of genetically encoded fluorescent molecules to study synapses inside living organisms.**

## Seeing one tree in a forest

In the mid-1800s, neuroscience experienced a scientific boom. The father of modern neuroscience, Santiago Ramon y Cajal, used Camillio Golgi’s silver stain to document complex morphologies that neurons have in the brain. This was because the stain would selectively label a few neurons and leave the rest invisible. His description of these neurons was also facilitated by his artistic talent, leaving us with beautiful renderings of the complex structures emanating from the cell bodies. Cajal demonstrated the power of sparse labelling, of being able to see individual cells with very high fidelity in a crowded field. From his observations, Cajal predicted that neurons would be separated by a gap, a hypothesis that went directly against Golgi’s model that neurons were reticulated, *i*.e., directly connected in the same fashion as our vascular system. We know now that both Cajal and Golgi were correct, and neurons form connections via both synapses and gap junctions.

Sadly, Cajal could not directly see synapses because his microscope lacked the required magnification. It was only later, as the field developed tools like immunohistochemistry, electron microscopy, and electrophysiology, that we were able to directly observe these subcellular junctions and measure their function. It was with the latter tools that we could measure synaptic plasticity and/or changes during development. However, observation of synapses still required fixation and either immunohistochemistry or electron microscopy.

## Mind the gap

**Fig. 1.**
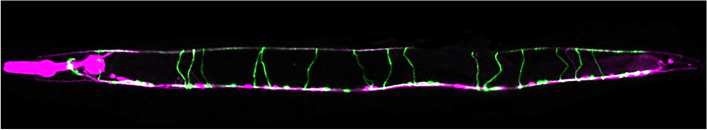
In this *C. elegans* micrograph, the D-type GABAergic motorneurons are labelled with green fluorescent protein, while the A-type cholinergic motorneurons are visualized using a red fluorescent protein (mCherry). The pharynx at the anterior is also labelled with mCherry as a transformation marker

After Douglas Prasher cloned the green fluorescent protein (GFP) gene, he sent the GFP plasmid to Marty Chalfie, among others. Marty expressed GFP in *Caenorhabditis elegans* and was able to see selective neurons inside living organisms [1]. Fusing the GFP coding sequence to the *C. elegans* gene that encodes synaptobrevin, a protein that resides in the plasma membrane of synaptic vesicles, allowed visualization of presynaptic contacts [2]. Yishi Jin selectively expressed the SNB-1::GFP chimera in the GABAergic motorneurons in *C. elegans* [3]. These neurons essentially “tile” the animal, resulting in a synaptic marker with single synapse resolution. Around the same time, Erik Jorgensen fused GFP to GABAergic receptors, which localized exquisitely to postsynaptic domains in muscles [4]. A little more than 100 years after Cajal had used sparse labelling to visualize individual neurons, we could now do the same for synapses (Fig. 1).

Very quickly, the power of these markers was demonstrated by the application of forward genetic screens. Demonstrating the power of genetics, we found completely unexpected results that informed our understanding of synaptic development. For example, the first published report of a mutant using a fluorescent synaptic marker described a role for, *lin-14*, a gene that was known to regulate developmental timing but had not been linked to synapse formation. Yishi Jin’s lab showed that, in a *lin-14-*dependent manner, specific neurons completely reorganized their wiring, including where synapses were located [3]. Although we knew synapses were structurally and functionally dynamic because of changes in activity, this was really the first observation that synapses changed their location within cells.

Later, dynamic synaptic proteins were observed in vertebrate neurons. Using a similar approach, Elly Nedivi and colleagues delivered genes encoding fluorescently labelled postsynaptic proteins to neurons by in utero electroporation. First focusing on inhibitory postsynaptic proteins (gephyrin) and later adding a label for excitatory postsynaptic proteins (PSD-95), they used two-photon microscopy to observe the dynamics of these proteins in dendritic terminals [5]. Using a genetic engineering strategy, they introduced the coding genes in reverse orientation relative to the promoter and flanked by LoxP site-specific recombination sites. Borrowing a “trick” from Cajal, they also provided the DNA for the fluorescent proteins at a high concentration, causing many cells to uptake the DNA, while providing Cre recombinase at a low level. This resulted in only a few spatially sparse neurons “flipping” the orientation of the coding genes and being labelled fluorescently. Amazingly, this revealed that some dendritic spines have proteins that cluster either excitatory or inhibitory receptors. They demonstrated that the inhibitory postsynaptic structures could move from dendritic spines to shafts but only in a subset of synapses.

## Mutations in synaptic proteins do not necessarily cause functional deficits

There have now been several forward genetic screens, primarily in invertebrates, using fluorescently labelled synaptic proteins. One of the striking patterns I have seen is that there is only a partial overlap of genes that affect synaptic activity and those that affect synapse formation/development. In *C. elegans*, the earliest genetic screens performed were those that affected motility (screening for uncoordinated/Unc movement) or affected body morphology. This made sense given that these were easily observed using a standard dissecting microscope. One of the first mutants isolated, *unc-2* (the second uncoordinated mutation recovered), encodes the α1 subunit of synaptic calcium channels required for exocytosis, and loss of function results in paralysis. However, the effects of removing *unc-2* on synaptic morphology are quite modest and only detectable in another mutant background [6]. Overall, while mutations in some of the genes that function in synaptic transmission result in synaptic morphology defects, not all do.

The converse is true as well. Several of the genes isolated in screens for synapse morphology defects cause no gross changes in behaviors. Not long after the role of *lin-14* in synapse remodelling was described, the Jin lab reported a mutation in a structural protein, called *syd-2/α-liprin* isolated in a *sy*napse *d*efective screen [7]. Subsequently, the Jin, Nonet, and Goodman labs simultaneously reported the cloning of the *r*egulator of *p*resynaptic *m*orphology 1 (*rpm-1*) in *C. elegans* [8, 9]. *The* Jin and Nonet labs isolated alleles of *rpm-1* in independent screens using the fluorescent vesicle marker in either GABAergic or mechanosensory neurons respectively. *C. elegans* lacking either *syd-2* or *rpm-1* have grossly wild-type movement, i.e., it is unlikely those mutants would have been isolated in a screen for motility defects.

However, when the Jin lab made double mutants of *rpm-1* and *syd-2*, they found that those animals have a very strong movement defect, appearing almost completely paralyzed. This allowed them to screen for genetic mutations that reverted these defects, i.e., restored movement. Unexpectedly, they isolated multiple alleles in the MAP kinase pathway, including *dlk-1* [10]. Like *unc-2*, loss of function in the MAP kinases were not obviously synapse defective, but they did suppress morphological defects in *rpm-1* mutant animals. Later, many different groups have shown that Dlk kinases are critical for neuronal regeneration in organisms where it occurs. In fact, loss of *rpm-1* function enhances regeneration, while removing *dlk-1* completely prevents regeneration. These data led to the hypothesis that synapse formation and neuronal regeneration may be mutually antagonistic.

## The future of fluorescent synaptic reporters

Here, I have provided a few of the intriguing results that have arisen from the early days of using fluorescent proteins to visualize synapses inside living organisms. Specifically, these tools enabled forward genetic screens that identified new players in the field of synapse biology that would have been missed by other approaches.

Where do we go from here? Well, the spectrum of available fluorescent molecules, and the modifications made to their function, is innumerable. For example, photoconvertible, photoactivatable, and reactive oxygen-, calcium-, or pH-sensitive molecules all enable us to see different kinds of biology in neurons and at the synapse. Much of the early work in synapse biology focused on the development of these structures, but we can also look at aging animals. What kinds of new lessons might we learn about neurodegenerative diseases in forward genetic screens? We might find specific synaptic morphologies that predict the onset of degenerative diseases, or we may identify genes where the loss of function results in synapse degeneration later in life, but the critical period for them to be intact is actually much earlier, perhaps even during development. Overall, many critical insights, far too many to fit in this brief commentary, have blossomed from the fertile ground of the first rounds of genetic screens using fluorescent synaptic markers. I believe Santiago Ramon y Cajal would be proud that his selective labelling approach, although accidental, is now being used to study a structure that he predicted but never observed.

## Data Availability

Not applicable.

## References

[1] Chalfie M, Tu Y, Euskirchen G, Ward WW, Prasher DC (1994). Green fluorescent protein as a marker for gene expression. Science.

[2] Nonet ML (1999). Visualization of synaptic specializations in live C. elegans with synaptic vesicle protein-GFP fusions. J Neurosci Methods.

[3] Hallam SJ, Jin Y (1998). lin-14 regulates the timing of synaptic remodelling in Caenorhabditis elegans. Nature.

[4] Bamber BA, Beg AA, Twyman RE, Jorgensen EM (1999). The Caenorhabditis elegans unc-49 locus encodes multiple subunits of a heteromultimeric GABA receptor. J Neurosci.

[5] Villa KL, Berry KP, Subramanian J, Cha JW, Chan OhW, Kwon HB (2016). Inhibitory synapses are repeatedly assembled and removed at persistent sites in vivo. Neuron.

[6] Caylor RC, Jin Y, Ackley BD (2013). The Caenorhabditis elegans voltage-gated calcium channel subunits UNC-2 and UNC-36 and the calcium-dependent kinase UNC-43/CaMKII regulate neuromuscular junction morphology. Neural Dev.

[7] Zhen M, Jin Y (1999). The liprin protein SYD-2 regulates the differentiation of presynaptic termini in C. elegans. Nature.

[8] Zhen M, Huang X, Bamber B, Jin Y (2000). Regulation of presynaptic terminal organization by C. elegans RPM-1, a putative guanine nucleotide exchanger with a RING-H2 finger domain. Neuron.

[9] Schaefer AM, Hadwiger GD, Nonet ML (2000). rpm-1, a conserved neuronal gene that regulates targeting and synaptogenesis in C. elegans. Neuron.

[10] Nakata K, Abrams B, Grill B, Goncharov A, Huang X, Chisholm AD (2005). Regulation of a DLK-1 and p38 MAP kinase pathway by the ubiquitin ligase RPM-1 is required for presynaptic development. Cell.

